# Editorial: Therapeutic neuromodulation for aging-related disorders associated with the autonomic nervous system

**DOI:** 10.3389/fnagi.2024.1399972

**Published:** 2024-03-26

**Authors:** Stephanie Chee Yee Tjen-A-Looi, Liang-Wu Fu, Shaista Malik, Richard E. Harris, Sae Uchida

**Affiliations:** ^1^Susan Samueli Integrative Health Institute, College of Health Sciences, University of California, Irvine, Irvine, CA, United States; ^2^Tokyo Metropolitan Institute for Geriatrics and Gerontology, Tokyo, Japan

**Keywords:** neurostimulation, electroacupuncture, exercise, hypertension, mild cognitive impairment

Neurostimulation is commonly used for pain management and works via neuromodulation involving neuroplasticity. Various types of neurostimulation have been used to manage low back pain. The chronic low back pain neurostimulation therapy could be delivered via percutaneous neuromodulation by electrodes, percutaneous electrical nerve stimulation by needles or electroacupuncture, transcutaneous electrical nerve stimulation, or manual acupuncture (Wu et al., 2018; Kim et al., 2020). In addition to pain, neurostimulation is also being considered for the treatment for disorders of the autonomic nervous system. Electroacupuncture and manual acupuncture are known to activate somatosensory fibers. These therapies influence the autonomic nervous system, neuronal pathways, and neurochemical systems and have the potential to alleviate pathological conditions associated with aging in which dysautonomia is prevalent (Zhou et al.) (Longhurst and Tjen-A-Looi, 2013). This Research Topic focuses on modulation of the aberrant physiological conditions through neuromodulation aside from pain.

Successful treatment of disorders associated with the autonomic nervous system involved in the regulation of blood pressure, glucose metabolism, cognitive function, and regulation of sympathetic tone is challenging. Importantly, aging is associated with various conditions including cognitive decline leading to dementia, cardiac events, hypertension, chronic inflammation, glucose intolerance, and chronic pain which affect quality of life, morbidity, and mortality. Many of these conditions have limited response to pharmacological treatments, and thus given the gap in treatment, non-pharmacological therapies are being increasingly investigated. Non-pharmacological treatments include lifestyle changes such as diet and exercise and various forms of acupuncture that lead to neuromuscular and sensory motor activation (Ahmad et al., 2019; Concha-Cisternas et al., 2023). This Research Topic includes studies demonstrating that non-pharmacological therapies such as electroacupuncture, acupuncture, and moderate exercise can potentially reduce high blood pressure, mild cognitive decline, and diabetes, a risk factor for heart failure.

For over 25 years, scientists have shown a number of mechanisms associated with central activity (neuronal pathways and circuitry, neurotransmitters, neuromodulators, and receptor subtypes) of the blood pressure lowering effect of electroacupuncture (Longhurst and Tjen-A-Looi, 2013; Fu et al., 2023). The proof-of-concept shows that electroacupuncture reduces both transient and sustained increases in sympathetic activity and blood pressure in animals and humans (Li et al., 2004, 2015, 2016; Tjen-A-Looi et al., 2004). In the aging population, hypertension associated with chronic low-grade inflammation and elevated activity of the sympathetic nervous system could be reduced with electroacupuncture. In a recent study, we have shown that electroacupuncture reduces both inflammation and sympathetic activity as evidenced by a decrease in cytokines and norepinephrine in hypertensive rats. Moreover, these two systems interact positively to reduce blood pressure in hypertensive subjects (Fu et al., 2023). Unique neurochemicals and receptor subtypes contribute to the actions of electroacupuncture. A recent study investigates the role of adenosine system during effect of electroacupuncture. The subtype Adenosine2a receptor also participates in the effects of electroacupuncture decreasing transient elevated blood pressure in normal rats (Malik et al., 2019). One article in this Research Topic investigates in salt sensitive hypertensive rats the role of the Adenosine2a receptor during the effects of electroacupuncture. Electroacupuncture stimulating the deep peroneal nerve reduces blood pressure by selectively activating Adenosine2a receptor in hypertensive subjects (Guo et al.).

Mild cognitive decline is associated with autonomic dysfunction (Nicolini et al., 2014). This cognitive impairment can progressively lead to dementia (Petersen, 2011), including Alzheimer's Disease, which is an increasingly prevalent condition in the aging population (Petersen et al., 2018). Cerebral white matter damage with gradual increased volume leading to mood disorders and cognitive memory impairment is associated with a higher risk of Alzheimer's Disease. White matter damage observed with magnetic resonance imaging for white matter hyperintensities (WMH) is also common in older adults (Otsuka et al.). Currently there are limited FDA approved pharmacological treatment to slow the progression of this impairment. The off-label medicines acetylcholinesterase inhibitors and N-methyl-D-aspartate receptor antagonists unfortunately induce side effects like gastrointestinal symptoms, confusion, dizziness, and headaches in these patients (Chin et al., 2022). Newly approved treatment, Leqembi can cause headaches, confusion, dizziness, and intracranial hemorrhage and edema. Non-pharmacological approaches such as acupuncture and exercise regimens are now being explored. Physical activity provides neuroprotective effects (including increased neurotrophic factors, decreased proinflammatory cytokines, and promoting neurocognitive function) in patients with mild cognitive impairment (Tsai et al., 2019). Acupuncture may also improve cognitive function in mild cognitive impairment (Yin et al., 2022, 2023). The two studies on mild cognitive and memory impairment in this Research Topic focus on an exercise intervention (Otsuka et al.) and a clinical study design with acupuncture therapy (Bao et al.). Daily maximal walking speed and moderate physical activity are associated with a smaller percent of WMH. Although acupuncture has been reported to modulate mild cognitive impairment, the mechanisms underlying this effect of acupuncture are unclear. The randomized controlled trial provides a protocol design investigating the effects of acupuncture on gut microbiota, mild cognitive impairment, and inflammatory cytokines in mild cognitive impaired patients and healthy subjects. Robust randomized controlled trials are urgently needed.

Diabetes increases the risk of heart failure while both conditions are influenced by chronic high sympathetic activity and hence investigations should explore the potential therapeutic effects of electroacupuncture and acupuncture (Zhou et al.). Persistent elevated sympathetic tone, activating the angiotensin-aldosterone system, results in increased cardiovascular activities such as heart rate, stroke volume and vasoconstriction. These processes lead to hypertension, a major risk factor for cardiac events including heart failure. Furthermore, diabetic cardiomyopathy includes impairment of cardiac structure and function in response to autonomic dysfunction and other factors (inflammation, oxidative stress, and others) (Ritchie and Abel, 2020). There is a lack of effective treatment for diabetes mellitus induced cardiac dysfunction. There is an urgent need for investigation of not just pharmacological but also non-pharmacological therapy for this condition. Although several preclinical and clinical studies report a blood glucose lowering effect of electroacupuncture, robust randomized blinded controlled clinical trials are warranted. Similarly, based on the heterogeneity of the heart failure studies with acupuncture intervention, the effectiveness of acupuncture for heart failure is inconclusive and more clinical trials are needed. This mini review offers a rationale for studies in effectiveness of acupuncture on the diabetic heart.

## Author contributions

ST-A-L: Writing – original draft, Writing – review & editing. L-WF: Writing – review & editing. SM: Writing – review & editing. RH: Writing – review & editing. SU: Writing – review & editing.
